# Optical scheme for generating hyperentanglement having photonic qubit and time-bin via quantum dot and cross-Kerr nonlinearity

**DOI:** 10.1038/s41598-018-19970-2

**Published:** 2018-02-07

**Authors:** Chang Ho Hong, Jino Heo, Min Sung Kang, Jingak Jang, Hyung Jin Yang

**Affiliations:** 1 Base Technology Division, National Security Research Institute, P.O. Box 1, Yuseong, Daejeon 34188 Republic of Korea; 2 College of Electrical and Computer Engineering, Chungbuk National University, Chungdae-ro 1, Seowon-Gu, Cheongju Republic of Korea; 3 Center for Quantum Information, Korea Institute of Science and Technology (KIST), Seoul, 136-791 Republic of Korea; 4 Department of Physics, Korea University, Sejong, 339-700 Republic of Korea

## Abstract

We design an optical scheme to generate hyperentanglement correlated with degrees of freedom (DOFs) via quantum dots (QDs), weak cross-Kerr nonlinearities (XKNLs), and linearly optical apparatuses (including time-bin encoders). For generating hyperentanglement having its own correlations for two DOFs (polarization and time-bin) on two photons, we employ the effects of optical nonlinearities using a QD (photon-electron), a parity gate (XKNLs), and time-bin encodings (linear optics). In our scheme, the first nonlinear multi-qubit gate utilizes the interactions between photons and an electron of QD confined in a single-sided cavity, and the parity gate (second gate) uses weak XKNLs, quantum bus, and photon-number-resolving measurement to entangle the polarizations of two photons. Finally, for efficiency in generating hyperentanglement and for the experimental implementation of this scheme, we discuss how the QD-cavity system can be performed reliably, and also discuss analysis of the immunity of the parity gate (XKNLs) against the decoherence effect.

## Introduction

Hyperentanglement is caused by the entanglement of a single system having correlations with several degrees of freedom (DOFs)^1–3^. Such hyperentanglement with various types of DOFs has been researched for enhancement of channel capacity^4,5^, including factors such as polarization and momentum^6^, polarization and orbital angular momentum^7^, and time-bin^8–10^. Thus, many quantum information processing schemes assisted by the advantages of hyperentanglement have been proposed, such as generation of hyperentanglement^10–13^, hyperentanglement concentrations^13–17^, purifications^18–22^ and distribution^23^ of hyperentanglement, analysis of hyperentangled Bell state^6,8,24–29^, and quantum communications^7,9,30–32^.

For the feasibility and efficiency of quantum information processing, schemes that could realize quantum information processing should be designed using physical resources and experimental implementation. From this point of view, quantum optics assisted by optical nonlinearities plays a significant role in experimentally realizing quantum information processing.

Quantum dots (QDs) inside micro-cavities^20,33–40^ can store quantum information long-term using long electron spin coherence time (${{\rm{T}}}_{2}^{{\rm{e}}}\sim \mu {\rm{s}}$)^41–43^ within a limited spin relaxation time (${{\rm{T}}}_{{\rm{1}}}^{{\rm{e}}}\sim {\rm{ms}}$)^44–46^. They are good candidates for efficient, feasible designs of quantum information processing schemes involving: quantum communications^20,40,47–50^, quantum controlled operations^36,51–55^, and the analysis and generation of entanglement^34,35,56,57^.

Cross-Kerr nonlinearities (XKNLs) have also been exploited for experimental implementation of diverse applications^10,28,58–68^ in quantum information processing. However, because the decoherence effect in the designed applications is induced due to loss of photons in optical fibers, the fidelity of these applications will decrease as the output state evolves into a mixed state^69–74^. Fortunately, by applying photon-number-resolving measurements^70,71,74^, and a displacement operator^70,71^ or quantum bus beams^74^ with the increasing amplitude of the coherent state, the decoherence effect can be made arbitrarily small^70,71,74^.

In this paper, we propose an optical scheme to generate hyperentanglement having its own correlations for two DOFs (polarization and time-bin) on two photons using the QD-cavity system, XKNLs, and linearly optical apparatuses (including time-bin encoders). For the generation of hyperentanglement, our scheme includes nonlinearly optical devices and linearly optical apparatuses, used as follows. **Nonlinear parts**: (1) The QD-cavity system is used in the interaction between photons and an excess electron of QD confined in a single-sided cavity^20,33–40^, and (2) The parity gate (polarization entanglexr) uses XKNLs, quantum bus beams, and photon-number-resolving measurement^60,67,72–74^. **Linear parts**: (1) The time-bin encoder uses circularly polarizing beam splitters (CPBS) and a delayed loop (DL), and (2) Another time-bin encoder uses Pockels cells (PCs)^10,16,20,23,75^. Consequently, we design a scheme for generating hyperentanglement, and herein report our analysis of the performance and efficiency of nonlinearly optical devices (QD-cavity system and parity gate using XKNLs) for practical experimental implementation.

## Nonlinear parts: QD-cavity system and parity gate using XKNLs

### The QD-cavity system

**Figure 1:**
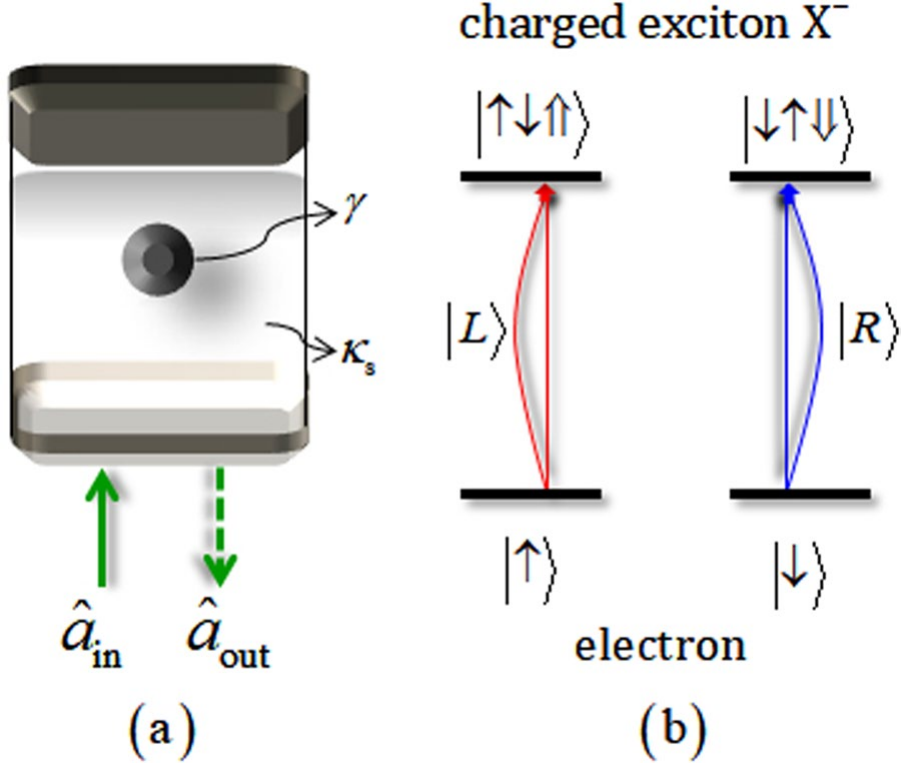
(**a**) A singly charged QD inside a single-sided cavity interacting with a photon, with side leakage rate (*κ*_*s*_) and decay rate (*γ*) of X^−^. (**b**) By the spin selection rule in the QD, the photon |*L*〉 (|*R*〉) drives the interaction as $|\uparrow \rangle \to |\uparrow \downarrow \Uparrow \rangle$ ($|\downarrow \rangle \to |\downarrow \uparrow \Downarrow \rangle$), where $|\uparrow \rangle \equiv |+1/2\rangle ,\,|\downarrow \rangle \equiv |-1/2\rangle$ are the spin states of the excess electron, and $|\Uparrow \rangle ,\,|\Downarrow \rangle \,({J}_{z}=+3/2,\,-3/2)$ represent heavy-hole spin states.

Here, we introduce the concept of a singly charged QD confined in a single-sided cavity. The QD-cavity system (single-sided cavity)^20,33–40^ consists of two GaAs/Al(Ga)As distributed Bragg reflectors (DBRs) and a transverse index guide for three-dimensional (3-D) confinement of light, where ${\hat{{a}}}_{{\rm{in}}}$ and ${\hat{{a}}}_{{\rm{out}}}$ are the input and output field operators, (*κ*_*s*_) is the side leakage rate, and the decay rate (*γ*) of a negatively charged exciton (X^−^) consists of two electrons bound to one hole^76^, as described in Fig. 1(a).

In Fig. 1(b), when the left circularly polarized photon $|L\rangle$ (right circularly polarized photon $|R\rangle$) is injected into the QD-cavity system, the polarized photon can create the spin state $|\uparrow \downarrow \Uparrow \rangle$ $(|\downarrow \uparrow \Downarrow \rangle )$ coupled to X^−^ in the spin state $|\uparrow \rangle$ $(|\downarrow \rangle )$ of the excess electron in QD according to the Pauli exclusion principle. By these spin-dependent optical transitions, the hot cavity ($|R\rangle |\downarrow \rangle$ or $|L\rangle |\uparrow \rangle$: the QD is coupled to the cavity) and the cold cavity ($|R\rangle |\uparrow \rangle$ or $|L\rangle |\downarrow \rangle$: the QD is uncoupled from the cavity) can induce different reflectances [|*r*_h_(*ω*)|, |*r*_0_(*ω*)|] and phases [*φ*_rh_(*ω*) = arg(*r*_h_(*ω*)), *φ*_r0_(*ω*) = arg(*r*_0_(*ω*))] of the reflected photon, as follows:

1 $$\begin{array}{rcl}{\rm{hot}}\,\mathrm{cavity}:\,{r}_{{\rm{h}}}(\omega ) & \equiv  & |{r}_{{\rm{h}}}(\omega )|{e}^{i{\phi }_{{\rm{rh}}}(\omega )}\\  & = & \frac{[i({\omega }_{c}-\omega )+\gamma /2]\,[i({\omega }_{c}-\omega )-\kappa /2+{\kappa }_{s}/2]+{g}^{2}}{[i({\omega }_{c}-\omega )+\gamma /2]\,[i({\omega }_{c}-\omega )+\kappa /2+{\kappa }_{s}/2]+{g}^{2}},\\ {\rm{cold}}\,\mathrm{cavity}:\,{r}_{{\rm{0}}}(\omega ) & \equiv  & |{r}_{{\rm{0}}}(\omega )|{e}^{i{\phi }_{{\rm{r0}}}(\omega )}\\  & = & \frac{i({\omega }_{c}-\omega )-\kappa /2+{\kappa }_{s}/2}{i({\omega }_{c}-\omega )+\kappa /2+{\kappa }_{s}/2},\end{array}$$

where *r*_h_(*ω*) and *r*_0_(*ω*) are the reflection coefficients, *ω*_*c*_ and *ω* are the frequencies of cavity mode and external field, and *κ* and *g* are the cavity decay rate of the cavity mode and the coupling strength (X^−^ ↔ cavity mode). In the weak excitation approximation^77^, we can obtain reflection coefficients for the steady state in Eq. 1 from the Heisenberg equation of motion with the ground state in QD (i.e., $\langle {\hat{\sigma }}_{Z}\rangle \approx -1$, ${\hat{\sigma }}_{Z}\hat{{a}}=-\hat{{a}}$), and the resonant interaction with ${\omega }_{c}={\omega }_{{{\rm{X}}}^{-}}$ (${\omega }_{{{\rm{X}}}^{-}}$: the frequency of the dipole transition of X^−^)^20,33–40^. Thus, the reflection operator $\hat{{\rm{R}}}(\omega )$, depending on the interaction between a polarized photon and the spin state of an electron inside a single-sided cavity, is given by

2 $$\begin{array}{rcl}\hat{{\rm{R}}}(\omega ) & = & |{r}_{{\rm{h}}}(\omega )|{e}^{i{\phi }_{{\rm{rh}}}(\omega )}(|R\rangle \langle R|\otimes |\downarrow \rangle \langle \downarrow |+|L\rangle \langle L|\otimes |\uparrow \rangle \langle \uparrow |)\\  &  & +|{r}_{0}(\omega )|{e}^{i{\phi }_{{\rm{r0}}}(\omega )}(|R\rangle \langle R|\otimes |\uparrow \rangle \langle \uparrow |+|L\rangle \langle L|\otimes |\downarrow \rangle \langle \downarrow |).\end{array}$$

Here, if the QD-cavity system having the small side-leakage rate, *κ*_*s*_ (${\kappa }_{s}\ll \kappa$), the strongly coupling strength $g\gg (\kappa ,\gamma )$ with small *γ* (about several μeV) for ${\omega }_{{{\rm{X}}}^{-}}={\omega }_{c}$, as shown in^33,78–80^, we can acquire |*r*_0_(*ω*)| = |*r*_h_(*ω*)| ≈ 1, *φ*_rh_(*ω*) = 0, and *φ*_r0_(*ω*) = ±*π*/2 through adjustment of the frequencies between the external field and cavity mode ($\omega -{\omega }_{c}=\mp \kappa /2$), and by omitting the leaky modes ${\hat{S}}_{{\rm{i}}{\rm{n}}}$ and vacuum noise $\hat{N}$^20,33–40^. Finally, when we take the experimental parameters *g*/*κ* = 2.4 and *κ*_*s*_ = 0 (negligible) with *ω* − *ω*_*c*_ = *κ*/2 and *γ*/*κ* = 0.1, the reflection operator $\hat{{\rm{R}}}(\omega )$ in Eq. 2 can be expressed as

3 $$\hat{{\rm{R}}}\approx (|R\rangle \langle R|\otimes |\downarrow \rangle \langle \downarrow |+|L\rangle \langle L|\otimes |\uparrow \rangle \langle \uparrow |)-i(|R\rangle \langle R|\otimes |\uparrow \rangle \langle \uparrow |+|L\rangle \langle L|\otimes |\downarrow \rangle \langle \downarrow |).$$

Subsequently, we will utilize this interaction of the QD-cavity system as a nonlinear optical device for the generation of hyperentanglement in our scheme.

### The polarization entangler (parity gate)

**Figure 2:**
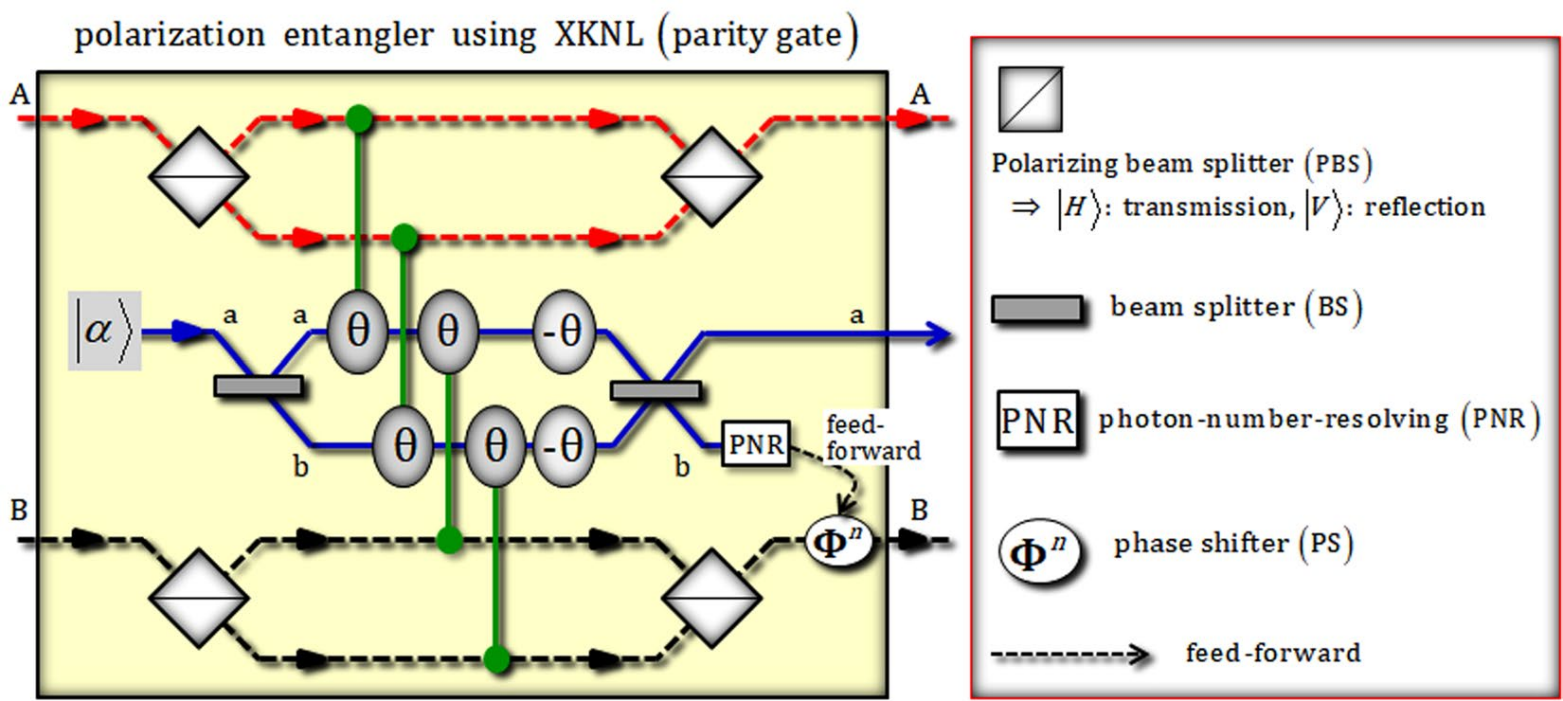
Plot representing the parity gate (polarization entangler) using XKNLs: a parity gate consists of XKNLs, quantum bus beams, photon-number-resolving measurement, and linearly optical apparatuses [PBSs, BSs, phase shifter (PS)]. The input state (product states of two photons) can be entangled between polarizations (single DOF) through this parity gate employing nonlinearly optical effects (XKNLs).

The XKNL’s Hamiltonian is given as *H*_*Kerr*_ = *ħχN*_1_*N*_2_, where *N*_*i*_ is photon-number operator and *χ* is the magnitude of nonlinearity in the Kerr medium. If we consider |*n*〉_1_ (photon state: *n* means photon-number) and |*α*〉_2_ (coherent state or probe beam), the state of photon-probe system is transformed to ${U}_{Kerr}{|n\rangle }_{1}{|\alpha \rangle }_{2}={e}^{i\theta {N}_{1}{N}_{2}}{|n\rangle }_{1}{|\alpha \rangle }_{2}={|n\rangle }_{1}{|\alpha {e}^{in\theta }\rangle }_{2}$ after the interactions in the Kerr medium, where *θ* = *χt* and *t* are the conditional phase-shift and the interaction time. Figure 2 shows a parity gate (polarization entangler), which can be operated using XKNLs, quantum bus beams, and photon-number-resolving measurement, to create entanglement between the polarizations of two photons. This parity gate^10,60,61,64,68,74^ is composed of four polarizing beam splitters (PBSs), four conditional phase-shifts (positive phases) in Kerr media, two linear phase-shift (minus phase), and two beam splitters (BSs) in quantum bus beams, as described in Fig. 2.

Here, let us assume the input state is ${|R\rangle }_{{\rm{A}}}{|R\rangle }_{{\rm{B}}}\otimes {|\alpha \rangle }^{{\rm{a}}}$ and define the relations of the circularly polarized states $\{|R\rangle ,|L\rangle \}$ and the linearly polarized states $\{|H\rangle ,|V\rangle \}$ as $|R\rangle \equiv (|H\rangle +|V\rangle )/\sqrt{2}$ and $|L\rangle \equiv (|H\rangle -|V\rangle )/\sqrt{2}$. After this input state passes through the PBSs, the Kerr media (XKNLs), and the BSs, the output state, $|{\phi }_{RR}\rangle$, of the parity gate is given by

4 $$\begin{array}{rcl}|{\phi }_{RR}\rangle  & = & |\alpha {\rangle }^{{\rm{a}}}\otimes \frac{1}{\sqrt{2}}\{\frac{1}{\sqrt{2}}({|H\rangle }_{{\rm{A}}}{|V\rangle }_{{\rm{B}}}+{|V\rangle }_{{\rm{A}}}{|H\rangle }_{{\rm{B}}})\}\otimes |0{\rangle }^{{\rm{b}}}\\  &  & +{|\alpha \cos \theta \rangle }^{{\rm{a}}}\otimes \frac{1}{\sqrt{2}}{e}^{-{(\alpha \sin \theta )}^{2}/2}\\  &  & \times \sum _{n=0}^{\infty }\tfrac{{(i\alpha \sin \theta )}^{n}}{\sqrt{n!}}\{\frac{1}{\sqrt{2}}({|V\rangle }_{{\rm{A}}}{|V\rangle }_{{\rm{B}}}+{(-1)}^{n}{|H\rangle }_{{\rm{A}}}{|H\rangle }_{{\rm{B}}})\}\otimes |n{\rangle }^{{\rm{b}}},\end{array}$$

where the BS transforms $|\alpha {\rangle }^{{\rm{a}}}|\beta {\rangle }^{{\rm{b}}}$ into ${|(\alpha +\beta )/\sqrt{2}\rangle }^{{\rm{a}}}{|(\alpha -\beta )/\sqrt{2}\rangle }^{{\rm{b}}}$, and $|\pm i\alpha \,\sin \,\theta \rangle ={e}^{-\frac{{(\alpha \sin \theta )}^{2}}{2}}\,\sum _{n=0}^{\infty }\frac{{(\pm i\alpha \sin \theta )}^{n}}{\sqrt{n!}}|n\rangle$ for *α* ∈ **R**. Then, we operate the photon-number-resolving measurement on the quantum bus beam of path b. When the measurement outcome is $|0{\rangle }^{{\rm{b}}}$ (dark detection), the output state will be ${(|H\rangle }_{{\rm{A}}}|V{\rangle }_{{\rm{B}}}+|V{\rangle }_{{\rm{A}}}|H{\rangle }_{{\rm{B}}})/\sqrt{2}$. Otherwise, ${|n\rangle }^{{\rm{b}}}\because n\ne 0$, the output state as ${(|V\rangle }_{{\rm{A}}}|V{\rangle }_{{\rm{B}}}+|H{\rangle }_{{\rm{A}}}|H{\rangle }_{{\rm{B}}})/\sqrt{2}$ via feed-forward [shifting of relative phase by PS (**Φ**^*n*^)] in accordance of measurement outcome *n* in Fig. 2. The error probability (P_err_), which is the probability of detection as $|0{\rangle }^{{\rm{b}}}$ (dark detection) in $|n{\rangle }^{{\rm{b}}}$ on path b, of this parity gate can be calculated by ${{\rm{P}}}_{{\rm{err}}}\approx {e}^{-{(\alpha \theta )}^{2}}/2$, where sin^2^*θ* ≈ *θ*^2^ for the strong magnitude of coherent state (probe beam: $\alpha \gg 1$) and $\theta \ll 1$. This means that the error probability, P_err_, can approach zero when increasing the magnitude of the probe beam or the conditional phase-shift (θ) of XKNL. However, the magnitude of XKNLs is tiny (very weak: θ ≈ 10^−18^)^81^, although the magnitude of the conditional phase-shift could be enhanced by electromagnetically induced transparency (EIT), θ ≈ 10^−2^ ^82,83^. Furthermore, as ref.^84^, it is experimentally difficult to implement the minus conditional phase-shift in the XKNL. Thus, we will utilize this polarization entangler (parity gate) employing quantum bus beams and photon-number-resolving measurement, which requires no negative XKNL (−θ) as a nonlinear optical device for the generation of hyperentanglement in our scheme.

## Scheme of generating hyperentanglement in two photons using a QD-cavity system, parity gate, and time-bin encoders

**Figure 3:**
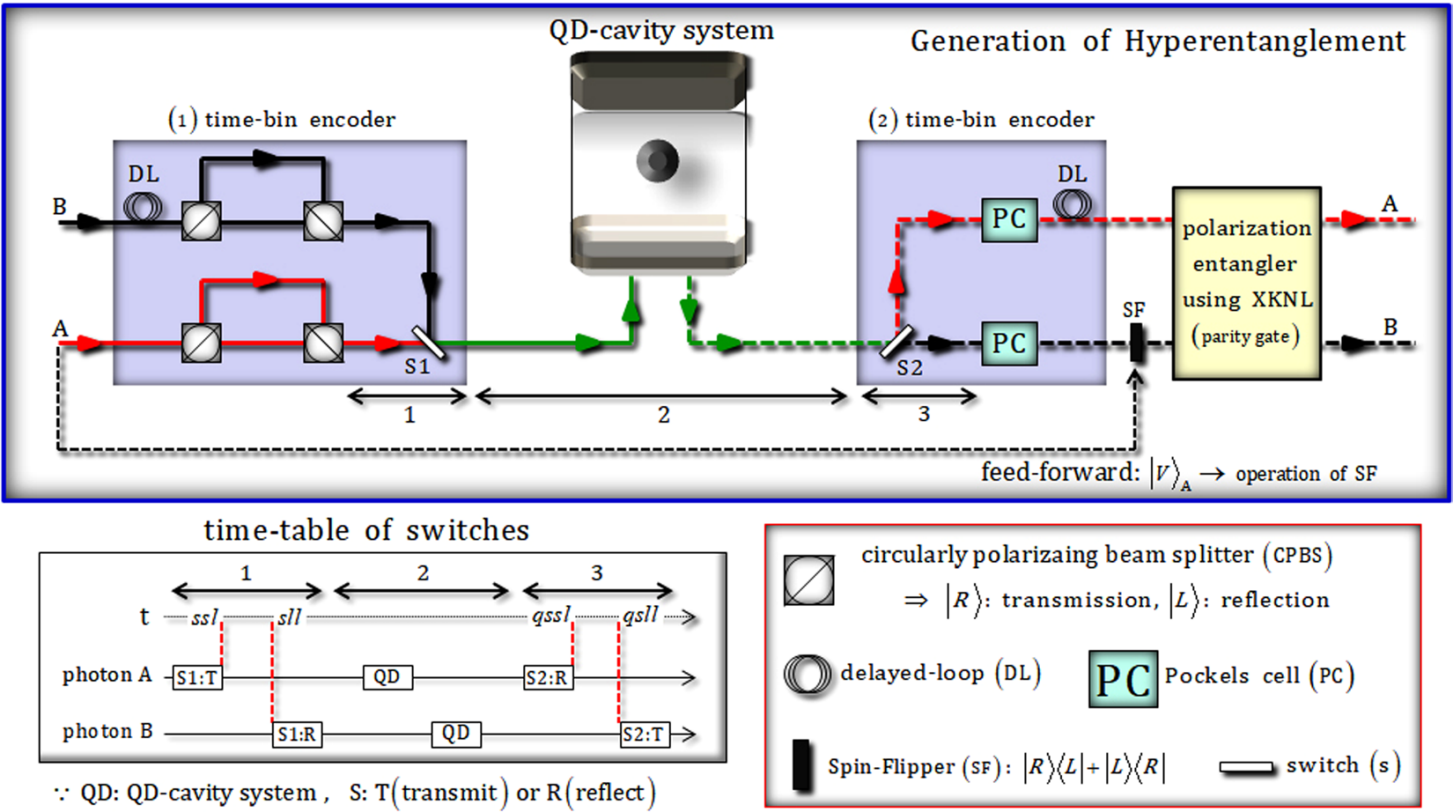
In our proposed scheme, the input state (product state) of two photons is encoded for two DOFs (polarization and time-bin) via (1) the time-bin encoder, which consists of CPBSs and delayed-loop (DL). Then, after the QD-cavity system (nonlinear part) in Sec. 2, (2) time-bin encoder based on PCs^10,16,20,23,75^ and DL can encode to polarizations (product state) and time-bin (entangled state) in two photons, respectively, where the switches (S1 and S2) in the time-bin encoders are operated according to a time-table. Subsequently, the polarization entangler parity gate (nonlinear part) using XKNLs completes to generate hyperentanglement having correlations for two DOFs (polarization and time-bin) in two photons (A and B).

We represent the generation of hyperentanglement having correlations for two DOFs (polarization and time-bin) via nonlinear optical devices (the QD-cavity system and parity gate) and linear optical apparatuses (including time-bin encoders), as shown in Fig. 3.

For a detailed description of this procedure, we assume the initial state of two photons as $|V{\rangle }_{{\rm{A}}}\otimes |H{\rangle }_{{\rm{B}}}$. If the polarization of photon A is a vertically polarized state, $|V\rangle$, then we perform the spin-flipper (SF) by feed-forward before the polarization entangler, as described in Fig. 3. After (1) time-bin encoder, the state $|{\phi }_{1}{\rangle }_{{\rm{AB}}}$ of two photons is given by

5 $$\begin{array}{c}{|V\rangle }_{{\rm{A}}}\otimes {|H\rangle }_{{\rm{B}}}\mathop{\longrightarrow }\limits^{(1)\,{\rm{t}}{\rm{i}}{\rm{m}}{\rm{e}}-{\rm{b}}{\rm{i}}{\rm{n}}\,{\rm{e}}{\rm{n}}{\rm{c}}{\rm{o}}{\rm{d}}{\rm{e}}{\rm{r}}}{|{\phi }_{1}\rangle }_{{\rm{A}}{\rm{B}}}\\ \begin{array}{ccc} & \Rightarrow  & \frac{1}{\sqrt{2}}({|R\rangle }_{{\rm{A}}}|sss\rangle -{|L\rangle }_{{\rm{A}}}|sls\rangle )\otimes \frac{1}{\sqrt{2}}({|R\rangle }_{{\rm{B}}}|lsl\rangle +{|L\rangle }_{{\rm{B}}}|lll\rangle ),\end{array}\end{array}$$

where the path length of photon B is longer than the path of photon A by DL (photon A: time interval *s* in the short length, photon B: time interval *l* in the long length) before CPBSs. Then, we can adjust the paths regarding two circular polarizations |*R*〉 and |*L*〉 of two photons by CPBSs, such as |*L*〉 obtaining the time interval *l* in the long length and |*R*〉 obtaining the time interval *s* over the short length. Finally, photon A (B) can obtain the time interval *s* (*l*) in the optical length, because the path length of photon A is shorter than the path of photon B before Switch 1 (S1).

Subsequently, we prepare a spin state, $|+{\rangle }_{{\rm{1}}}\equiv ({|\uparrow \rangle }_{{\rm{1}}}+{|\downarrow \rangle }_{{\rm{1}}})/\sqrt{2}$, of electron 1 in QD. After the photons A and B pass through S1 according to the time-table of switches in Fig. 3, they interact with the QD-cavity system, in sequence. The output state $|{\phi }_{2}{\rangle }_{{\rm{1AB}}}$ of electron 1 and two photons, after the interactions between photons and an electron of QD as described in Eq. 3, is transformed as below

6 $$\begin{array}{c}{|+\rangle }_{1}\otimes {|{\phi }_{1}\rangle }_{{\rm{A}}{\rm{B}}}\mathop{\longrightarrow }\limits^{{\rm{Q}}{\rm{D}}-{\rm{c}}{\rm{a}}{\rm{v}}{\rm{i}}{\rm{t}}{\rm{y}}\,{\rm{s}}{\rm{y}}{\rm{s}}{\rm{t}}{\rm{e}}{\rm{m}}}{|{\phi }_{2}\rangle }_{1{\rm{A}}{\rm{B}}}\\ \begin{array}{ccc} & \Rightarrow  & \frac{i}{\sqrt{2}}[{|+\rangle }_{1}\otimes \frac{-1}{\sqrt{2}}({|R\rangle }_{{\rm{A}}}{|qsss\rangle }_{{\rm{A}}}\otimes {|L\rangle }_{{\rm{B}}}{|qlll\rangle }_{{\rm{B}}}-{|L\rangle }_{{\rm{A}}}{|qssl\rangle }_{{\rm{A}}}\otimes {|R\rangle }_{{\rm{B}}}{|qsll\rangle }_{{\rm{B}}})\\  &  & +i{|-\rangle }_{1}\otimes \frac{-1}{\sqrt{2}}({|R\rangle }_{{\rm{A}}}{|qsss\rangle }_{{\rm{A}}}\otimes {|R\rangle }_{{\rm{B}}}{|qsll\rangle }_{{\rm{B}}}+{|L\rangle }_{{\rm{A}}}{|qssl\rangle }_{{\rm{A}}}\otimes {|L\rangle }_{{\rm{B}}}{|qlll\rangle }_{{\rm{B}}})],\end{array}\end{array}$$

where we consider that the total time interval of the path length and the interaction time of the QD-cavity is *q*, and also that $|-{\rangle }_{{\rm{1}}}\equiv ({|\uparrow \rangle }_{{\rm{1}}}-{|\downarrow \rangle }_{{\rm{1}}})/\sqrt{2}$, and $|qlsl\rangle =|qsll\rangle ,\,|qsls\rangle =|qssl\rangle$.

In (2) the time-bin encoder, after the photons A and B pass through S2 due to the time-table of switches in Fig. 3, photon A (B) can obtain the time interval *l* (*s*) in the optical length because the path length of photon A is longer than the path of photon B before PCs. Then we utilize PCs, which affect a bit-flip operation on the polarization at a specific time^10,16,20,23,75^, to flip the polarizations of the photons. Here the action of the PCs flips the polarizations of the photon A at time-bin *qssll*(=*lqssl*), and the photon B at time-bin *qslll*(=*sqlll*). After PCs, the photon A (B) can acquire the time interval *l* (*s*) in the optical length because the path length of photon B is shorter than the path of photon A by DL. Thus, after passing the (2) time-bin encoder, the output state is given by

7 $$\begin{array}{c}{|{\phi }_{2}\rangle }_{1{\rm{A}}{\rm{B}}}\mathop{\longrightarrow }\limits^{(2)\,{\rm{t}}{\rm{i}}{\rm{m}}{\rm{e}}-{\rm{b}}{\rm{i}}{\rm{n}}\,{\rm{e}}{\rm{n}}{\rm{c}}{\rm{o}}{\rm{d}}{\rm{e}}{\rm{r}},\,{\rm{S}}{\rm{F}}\,({\rm{p}}{\rm{h}}{\rm{o}}{\rm{t}}{\rm{o}}{\rm{n}}\,{\rm{B}}):\,{\rm{f}}{\rm{e}}{\rm{e}}{\rm{d}}-{\rm{f}}{\rm{o}}{\rm{r}}{\rm{w}}{\rm{a}}{\rm{r}}{\rm{d}}}{|{\phi ^{\prime} }_{2}\rangle }_{{\rm{A}}{\rm{B}}1}\\ \begin{array}{c}\begin{array}{ccc} & \Rightarrow  & {|R\rangle }_{{\rm{A}}}{|L\rangle }_{{\rm{B}}}\otimes \frac{-i}{2}[|+{\rangle }_{1}({|llqsss\rangle }_{{\rm{A}}}{|ssqlll\rangle }_{{\rm{B}}}-{|llqssl\rangle }_{{\rm{A}}}{|ssqsll\rangle }_{{\rm{B}}})\\  &  & +|-{\rangle }_{1}({|llqsss\rangle }_{{\rm{A}}}{|ssqsll\rangle }_{{\rm{B}}}+{|llqssl\rangle }_{{\rm{A}}}{|ssqlll\rangle }_{{\rm{B}}})]\\  & \equiv  & {|R\rangle }_{{\rm{A}}}{|L\rangle }_{{\rm{B}}}\otimes [\frac{-i}{\sqrt{2}}|+{\rangle }_{1}\frac{1}{\sqrt{2}}({|s^{\prime} \rangle }_{{\rm{A}}}{|l^{\prime} \rangle }_{{\rm{B}}}-{|l^{\prime} \rangle }_{{\rm{A}}}{|s^{\prime} \rangle }_{{\rm{B}}})\\  &  & +\frac{-i}{\sqrt{2}}|-{\rangle }_{1}\frac{1}{\sqrt{2}}({|s^{\prime} \rangle }_{{\rm{A}}}{|s^{\prime} \rangle }_{{\rm{B}}}+{|l^{\prime} \rangle }_{{\rm{A}}}{|l^{\prime} \rangle }_{{\rm{B}}})],\end{array}\end{array}\end{array}$$

where we define as $$|{s}{^{\prime} }\rangle \equiv |{llqsss}\,{\rm{or}}\,{ssqsll}\rangle =|{l}\times 2+{s}\times 3\rangle $$ (short interval) and $$|{l}{^{\prime} }\rangle \equiv {|}\mathrm{ssqlll}\,{\rm{or}}\,{llqssl}\rangle =$$$$|{l}\times 3+{s}\times 2\rangle $$ (long interval). Because the initial state of photon A is a vertically polarized state, |*V*〉_A_, we perform SF to photon B by feed-forward $${(|R\rangle }_{{\rm{B}}}\to |L{\rangle }_{{\rm{B}}})$$, as described in Fig. 3 before the polarization entangler.

In the polarization entangler (parity gate) using XKNLs, as described in Sec. 2, the output state $|{\phi }_{{\rm{f}}}{\rangle }_{{\rm{1AB}}}$, according to Eq. 4, is transformed, as follows:

8 $$\begin{array}{c}{|{\phi }_{3}\rangle }_{{\rm{A}}{\rm{B}}1}\mathop{\longrightarrow }\limits^{{\rm{p}}{\rm{o}}{\rm{l}}{\rm{a}}{\rm{r}}{\rm{i}}{\rm{z}}{\rm{a}}{\rm{t}}{\rm{i}}{\rm{o}}{\rm{n}}\,{\rm{e}}{\rm{n}}{\rm{t}}{\rm{a}}{\rm{n}}{\rm{g}}{\rm{l}}{\rm{e}}{\rm{r}}}{|{\phi }_{{\rm{f}}}\rangle }_{1{\rm{A}}{\rm{B}}}\\ \begin{array}{ccc} & \Rightarrow  & \frac{-i}{\sqrt{2}}(|+{\rangle }_{1}\otimes {|{{\boldsymbol{\Psi }}}_{{\rm{T}}}^{-}\rangle }_{{\rm{A}}{\rm{B}}}+|-{\rangle }_{1}\otimes {|{{\boldsymbol{\Phi }}}_{{\rm{T}}}^{+}\rangle }_{{\rm{A}}{\rm{B}}})\otimes [{|\alpha \rangle }^{{\rm{a}}}\otimes \frac{-1}{\sqrt{2}}\{\frac{1}{\sqrt{2}}({|H\rangle }_{{\rm{A}}}{|V\rangle }_{{\rm{B}}}\\  &  & -{|V\rangle }_{{\rm{A}}}{|H\rangle }_{{\rm{B}}})\}\otimes |0{\rangle }^{{\rm{b}}}\\  &  & +{|\alpha \cos \theta \rangle }^{{\rm{a}}}\otimes \frac{-1}{\sqrt{2}}{e}^{-{(\alpha \sin \theta )}^{2}/2}\sum _{n=0}^{{\rm{\infty }}}\frac{{(i\alpha \sin \theta )}^{n}}{\sqrt{n!}}\{\frac{1}{\sqrt{2}}(-{|V\rangle }_{{\rm{A}}}{|V\rangle }_{{\rm{B}}}\\  &  & +{(-1)}^{n}{|H\rangle }_{{\rm{A}}}{|H\rangle }_{{\rm{B}}})\}\otimes |n{\rangle }^{{\rm{b}}}],\end{array}\end{array}$$

where we define the four types of polarization state (two photons), and the time-bin state (time interval), as follows:

9 $$\begin{array}{ll}({\rm{Polarization}}) & {|{{\boldsymbol{\Psi }}}_{{\rm{P}}}^{\pm }\rangle }_{{\rm{AB}}}\equiv \frac{1}{\sqrt{2}}({|{H}\rangle }_{{\rm{A}}}{|{V}\rangle }_{{\rm{B}}}\pm {|{V}\rangle }_{{\rm{A}}}{|{H}\rangle }_{{\rm{B}}}),\\  & {|{{\boldsymbol{\Phi }}}_{{\rm{P}}}^{\pm }\rangle }_{{\rm{AB}}}\equiv \frac{1}{\sqrt{2}}({|{H}\rangle }_{{\rm{A}}}{|{H}\rangle }_{{\rm{B}}}\pm {|{V}\rangle }_{{\rm{A}}}{|{V}\rangle }_{{\rm{B}}}),\\ (\mathrm{Time}-\mathrm{bin}) & {|{{\boldsymbol{\Psi }}}_{{\rm{T}}}^{\pm }\rangle }_{{\rm{AB}}}\equiv \frac{1}{\sqrt{2}}({|{s}{^{\prime} }\rangle }_{{\rm{A}}}{|{l}{^{\prime} }\rangle }_{{\rm{B}}}\pm {|{l}{^{\prime} }\rangle }_{{\rm{A}}}{|{s}{^{\prime} }\rangle }_{{\rm{B}}}),\\  & {|{{\boldsymbol{\Phi }}}_{{\rm{T}}}^{\pm }\rangle }_{{\rm{AB}}}\equiv \frac{1}{\sqrt{2}}({|{s}{^{\prime} }\rangle }_{{\rm{A}}}{|{s}{^{\prime} }\rangle }_{{\rm{B}}}\pm {|{l}{^{\prime} }\rangle }_{{\rm{A}}}{|{l}{^{\prime} }\rangle }_{{\rm{B}}}),\end{array}$$

where {|*H*〉, |*V*〉} is the horizontal, vertical polarization on the photon, and {|*s*′〉, |*l*′〉}is short interval, long interval due to the path length of the photon. Subsequently, if the measurement outcomes of the QD-cavity system (electron 1), and the quantum bus beam on path b by the photon-number-resolving measurement are ${\{|+{\rangle }_{1},|n\rangle }^{{\rm{b}}}\because n\ne 0\}$, then the final hyperentangled state having its own correlations for two DOFs (polarization and time-bin) on two photons will be ${|{{\boldsymbol{\Phi }}}_{{\rm{P}}}^{-}\rangle }_{{\rm{AB}}}\otimes {|{{\boldsymbol{\Psi }}}_{{\rm{T}}}^{-}\rangle }_{{\rm{AB}}}$ where the output state ${(-|V\rangle }_{{\rm{A}}}|V{\rangle }_{{\rm{B}}}+{(-1)}^{n}|H{\rangle }_{{\rm{A}}}|H{\rangle }_{{\rm{B}}})/\sqrt{2}$ in Eq. 8 is transformed to ${|{{\boldsymbol{\Phi }}}_{{\rm{P}}}^{-}\rangle }_{{\rm{AB}}}$ via feed-forward [shifting of relative phase by PS (**Φ**^*n*^)] according to result *n*. Table 1 shows that the possible hyperentangled states (having two DOFs) of two photons can be generated in accordance with the preparation of the initial states (product state), and the measurement outcomes of electron spin 1 inside the QD-cavity system, and the quantum bus beam on path b through the photon-number-resolving measurement.

**Table 1:** The generated hyperentanglement having its own correlations for two DOFs (polarization and time-bin) on two photons, according to the initial state, the results of electron spin 1 in QD and photon-number-resolving measurement of the quantum bus beam on path b.

The initial state of two photons (product state)	Result of electron 1	Result of photon-number-resolving measurement	Hyperentanglement of two photons for two DOFs
$|H{\rangle }_{{\rm{A}}}\otimes \frac{1}{\sqrt{2}}{(|R\rangle }_{{\rm{B}}}\pm |L{\rangle }_{{\rm{B}}})$	|+〉_1_	|0〉^b^	${|{{\boldsymbol{\Psi }}}_{{\rm{P}}}^{+}\rangle }_{{\rm{AB}}}\otimes {|{{\boldsymbol{\Psi }}}_{{\rm{T}}}^{\pm }\rangle }_{{\rm{AB}}}$
		|*n*〉^b^	${|{{\boldsymbol{\Phi }}}_{{\rm{P}}}^{+}\rangle }_{{\rm{AB}}}\otimes {|{{\boldsymbol{\Psi }}}_{{\rm{T}}}^{\pm }\rangle }_{{\rm{AB}}}$
	|−〉_1_	|0〉^b^	${|{{\boldsymbol{\Psi }}}_{{\rm{P}}}^{+}\rangle }_{{\rm{AB}}}\otimes {|{{\boldsymbol{\Phi }}}_{{\rm{T}}}^{\mp }\rangle }_{{\rm{AB}}}$
		|*n*〉^b^	${|{{\boldsymbol{\Phi }}}_{{\rm{P}}}^{+}\rangle }_{{\rm{AB}}}\otimes {|{{\boldsymbol{\Phi }}}_{{\rm{T}}}^{\mp }\rangle }_{{\rm{AB}}}$
$|V{\rangle }_{{\rm{A}}}\otimes \frac{1}{\sqrt{2}}{(|R\rangle }_{{\rm{B}}}\pm |L{\rangle }_{{\rm{B}}})$	|+〉_1_	|0〉^b^	${|{{\boldsymbol{\Psi }}}_{{\rm{P}}}^{-}\rangle }_{{\rm{AB}}}\otimes {|{{\boldsymbol{\Psi }}}_{{\rm{T}}}^{\mp }\rangle }_{{\rm{AB}}}$
		|*n*〉^b^	${|{{\boldsymbol{\Phi }}}_{{\rm{P}}}^{-}\rangle }_{{\rm{AB}}}\otimes {|{{\boldsymbol{\Psi }}}_{{\rm{T}}}^{\mp }\rangle }_{{\rm{AB}}}$
	|−〉_1_	|0〉^b^	${|{{\boldsymbol{\Psi }}}_{{\rm{P}}}^{-}\rangle }_{{\rm{AB}}}\otimes {|{{\boldsymbol{\Phi }}}_{{\rm{T}}}^{\pm }\rangle }_{{\rm{AB}}}$
		|*n*〉^b^	${|{{\boldsymbol{\Phi }}}_{{\rm{P}}}^{-}\rangle }_{{\rm{AB}}}\otimes {|{{\boldsymbol{\Phi }}}_{{\rm{T}}}^{\pm }\rangle }_{{\rm{AB}}}$

So far, we designed a scheme to generate hyperentanglement having its own correlations for two DOFs (polarization and time-bin) utilizing nonlinear optical devices (the QD-cavity system and the parity gate using XKNLs), and the linear optical apparatuses (time-bin encoders). In our schemes, the important parts (nonlinear optical devices) are the QD-cavity system and polarization entangler (parity gate) utilizing XKNLs, quantum bus beams, and the photon-number-resolving measurement. Therefore, we will analyze the performance and efficiency of the nonlinear optical devices for the experimental implementation in practice.

## Analysis of nonlinear parts: QD-cavity system and parity gate using XKNLs

### The QD-cavity system

The QD-cavity system interactions, which can induce difference in the reflectances and phases of the reflected photon according to the hot cavity (coupled) and cold cavity (uncoupled) conditions in Eq. 1, are significantly utilized for the reliable performance of our scheme. Thus, we should analyze the actual efficiency and experimental performance of these interactions of the QD-cavity system. For the reflection coefficient *r*(*ω*) with the noise *N*(*ω*) and leakage *S*(*ω*) coefficients, the Heisenberg equations of motion for a cavity field operator $(\hat{{a}})$, a dipole operator $({\hat{\sigma }}_{-})$ of X^−^, and the input-output relations^77^, are given by

10 $$\begin{array}{rcl}\frac{d\hat{{a}}}{dt} & = & -[i({\omega }_{c}-\omega )+\frac{\kappa }{2}+\frac{{\kappa }_{s}}{2}]\hat{{a}}-g{\hat{\sigma }}_{-}-\sqrt{\kappa }{\hat{{a}}}_{{\rm{in}}}-\sqrt{{\kappa }_{s}}{\hat{S}}_{{\rm{in}}},\\ \frac{d{\hat{\sigma }}_{-}}{dt} & = & -[i({\omega }_{{{\rm{X}}}^{-}}-\omega )+\frac{\gamma }{2}]{\hat{\sigma }}_{-}-g{\hat{\sigma }}_{Z}\hat{{a}}+\sqrt{\gamma }{\hat{\sigma }}_{Z}\hat{N},\\ {\hat{{a}}}_{{\rm{out}}} & = & {\hat{{a}}}_{{\rm{in}}}+\sqrt{\kappa }\hat{{a}},\end{array}$$

where ${\hat{S}}_{{\rm{in}}}$ is an input field operator from leaky modes due to sideband leakage and absorption, and $\hat{N}$ is the vacuum noise operator for ${\hat{\sigma }}_{-}$. In the weak excitation approximation^77^ and the ground state in QD (i.e., $\langle {\hat{\sigma }}_{Z}\rangle \approx -1$, ${\hat{\sigma }}_{Z}\hat{{a}}=-\hat{{a}}$) with ${\omega }_{c}={\omega }_{{{\rm{X}}}^{-}}$^20,33–40^, we can calculate the reflection coefficient *R*(*ω*) with the noise *N*(*ω*) and leakage *S*(*ω*) coefficients, as follows:

11 $$\begin{array}{rcl}R(\omega ) & = & \frac{[i({\omega }_{c}-\omega )+\gamma /2]\,[i({\omega }_{c}-\omega )-\kappa /2+{\kappa }_{s}/2]+{g}^{2}}{[i({\omega }_{c}-\omega )+\gamma /2]\,[i({\omega }_{c}-\omega )+\kappa /2+{\kappa }_{s}/2]+{g}^{2}},\\ N(\omega ) & = & \frac{\sqrt{\gamma \kappa }g}{[i({\omega }_{c}-\omega )+\gamma /2]\,[i({\omega }_{c}-\omega )+\kappa /2+{\kappa }_{s}/2]+{g}^{2}},\\ S(\omega ) & = & \frac{-\sqrt{{\kappa }_{s}\kappa }[i({\omega }_{c}-\omega )+\gamma /2]}{[i({\omega }_{c}-\omega )+\gamma /2]\,[i({\omega }_{c}-\omega )+\kappa /2+{\kappa }_{s}/2]+{g}^{2}}.\end{array}$$

**Figure 4:**
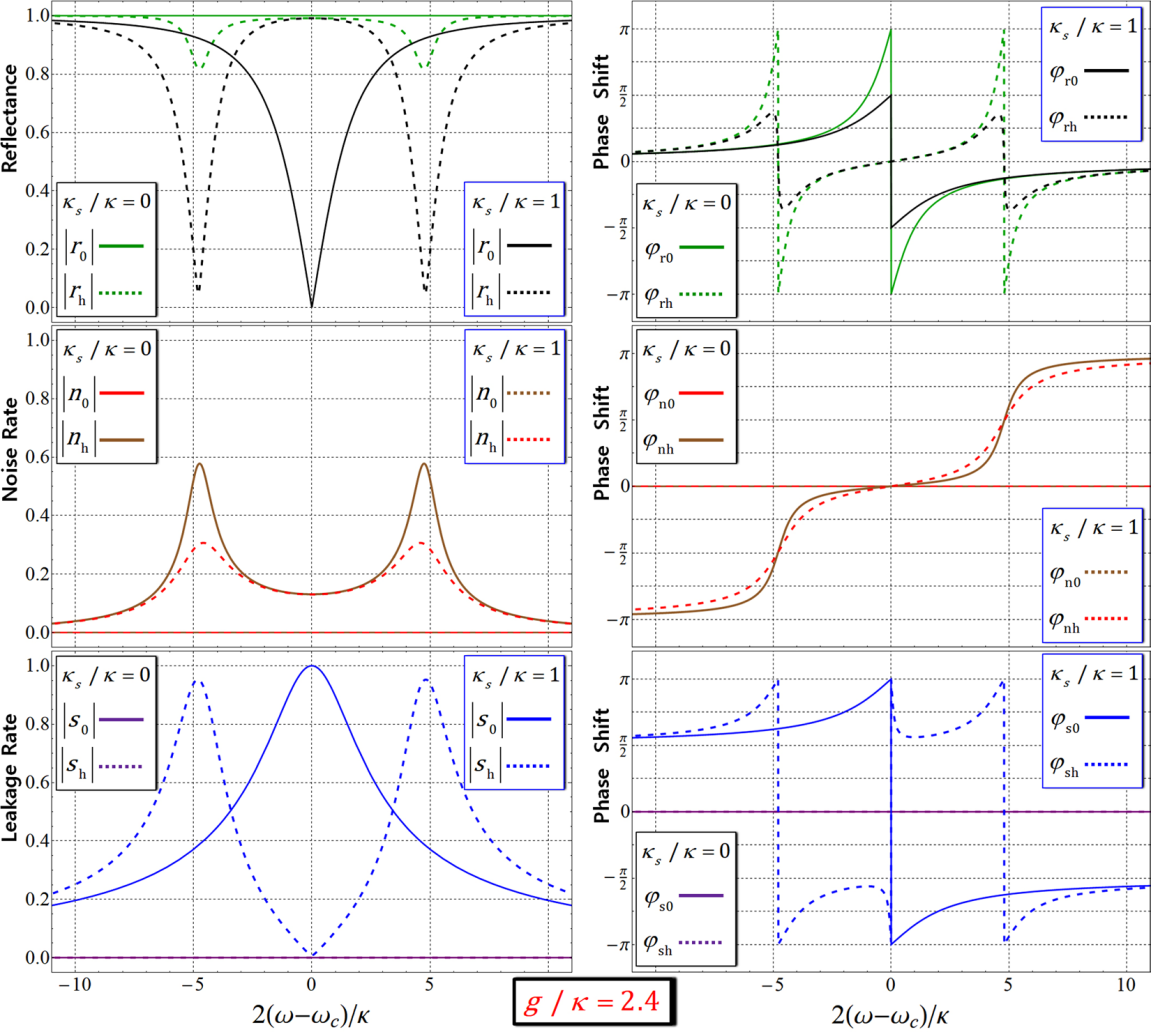
The left figures represent the reflectances [|*r*_h_(*ω*)|, |*r*_0_(*ω*)|], the noise rates [|*n*_h_(*ω*)|, |*n*_0_(*ω*)|], and the leakage rates [|*s*_h_(*ω*)|, |*s*_0_(*ω*)|], and also the right figures represent the phase shifts [*φ*_rh_(*ω*), *φ*_r0_(*ω*), *φ*_nh_(*ω*), *φ*_n0_(*ω*), *φ*_sh_(*ω*), *φ*_s0_(*ω*)] for frequency detuning 2(*ω* − *ω*_*c*_)/*κ*, according to the difference in the side leakage rates (*κ*_*s*_ = 0 and *κ*_*s*_ = 1.0*κ*). In these plots, we take the parameters as *g*/*κ* = 2.4 and *γ*/*κ* = 0.1 (*g* > (*κ*, *γ*)) with ${\omega }_{{{\rm{X}}}^{-}}={\omega }_{c}$^17,33–40^.

Considering the hot (*g* ≠ 0) and cold (*g* = 0) cavities, the reflection coefficients [*r*_h_(*ω*) and *r*_0_(*ω*)] are represented in Eq. 1. In addition, the noise [*n*_h_(*ω*) and *n*_0_(*ω*)] rates; and leakage [*s*_h_(*ω*) and *s*_0_(*ω*)] coefficients are given by

12 $$\begin{array}{rcl}{\rm{hot}}\,\mathrm{cavity}:\,{n}_{{\rm{h}}}(\omega ) & \equiv  & |{n}_{{\rm{h}}}(\omega )|{e}^{i{\phi }_{{\rm{nh}}}(\omega )}=\tfrac{\sqrt{\gamma \kappa }g}{[i({\omega }_{c}-\omega )+\gamma /2]\,[i({\omega }_{c}-\omega )+\kappa /2+{\kappa }_{s}/2]+{g}^{2}}=N(\omega ),\\ {\rm{cold}}\,\mathrm{cavity}:\,{n}_{{\rm{0}}}(\omega ) & \equiv  & |{n}_{{\rm{0}}}(\omega )|{e}^{i{\phi }_{{\rm{n0}}}(\omega )}=0,\\ {\rm{hot}}\,\mathrm{cavity}:\,{s}_{{\rm{h}}}(\omega ) & \equiv  & |{s}_{{\rm{h}}}(\omega )|{e}^{i{\phi }_{{\rm{sh}}}(\omega )}=\tfrac{-\sqrt{{\kappa }_{s}\kappa }[i({\omega }_{c}-\omega )+\gamma /2]}{[i({\omega }_{c}-\omega )+\gamma /2]\,[i({\omega }_{c}-\omega )+\kappa /2+{\kappa }_{s}/2]+{g}^{2}}=S(\omega ),\\ {\rm{cold}}\,\mathrm{cavity}:\,{s}_{{\rm{0}}}(\omega ) & \equiv  & |{s}_{{\rm{0}}}(\omega )|{e}^{i{\phi }_{{\rm{s0}}}(\omega )}=\tfrac{-\sqrt{{\kappa }_{s}\kappa }}{i({\omega }_{c}-\omega )+\kappa /2+{\kappa }_{s}/2},\end{array}$$

where [|*n*_h_(*ω*)|, *φ*_nh_(*ω*): hot cavity] and [|*n*_0_(*ω*)|, *φ*_n0_(*ω*): cold cavity] are the noise rates and the phase shifts, and [|*s*_h_(*ω*)|, *φ*_sh_(*ω*): hot cavity] and [|*s*_0_(*ω*)|, *φ*_s0_(*ω*): cold cavity] are the leakage rates and the phase shifts. Thus, the reflection operator $\hat{{\rm{R}}}(\omega )$ in Eq. 2 after the reflection from the QD-cavity system should be revised as

13 $$\begin{array}{rcl}{\hat{{\rm{R}}}}_{{\rm{P}}}(\omega ) & = & [|{r}_{{\rm{h}}}(\omega )|{e}^{i{\phi }_{{\rm{rh}}}(\omega )}+|{n}_{{\rm{h}}}(\omega )|{e}^{i{\phi }_{{\rm{nh}}}(\omega )}+|{s}_{{\rm{h}}}(\omega )|{e}^{i{\phi }_{{\rm{sh}}}(\omega )}]\\  &  & \times (|R\rangle \langle R|\otimes |\downarrow \rangle \langle \downarrow |+|L\rangle \langle L|\otimes |\uparrow \rangle \langle \uparrow |)\\  &  & +[|{r}_{{\rm{0}}}(\omega )|{e}^{i{\phi }_{{\rm{r0}}}(\omega )}+|{s}_{{\rm{0}}}(\omega )|{e}^{i{\phi }_{{\rm{s0}}}(\omega )}]\\  &  & \times (|R\rangle \langle R|\otimes |\uparrow \rangle \langle \uparrow |+|L\rangle \langle L|\otimes |\downarrow \rangle \langle \downarrow |),\end{array}$$

where ${n}_{{\rm{0}}}(\omega )\equiv |{n}_{{\rm{0}}}(\omega )|{e}^{i{\phi }_{{\rm{n0}}}(\omega )}=0$ from Eq. 12. When the QD-cavity system having the experimental parameters as *g*/*κ* = 2.4 and *γ*/*κ* = 0.1 (*g* > (*κ*, *γ*)) for small *γ* (about several μeV)^33,78–80^ with ${\omega }_{{{\rm{X}}}^{-}}={\omega }_{c}$, the reflectances and the phase shifts (|*r*_h_|, *φ*_rh_: hot cavity) and (|*r*_0_|, *φ*_r0_: cold cavity), the noise rates and the phase shifts (|*n*_h_|, *φ*_nh_: hot cavity) and (|*n*_0_|, *φ*_n0_: cold cavity), and the leakage rates and the phase shifts (|*s*_h_|, *φ*_sh_: hot cavity) and (|*s*_0_|, *φ*_s0_: cold cavity) are plotted for frequency detuning 2(*ω* − *ω*_*c*_)/*κ* according to the difference in side-leakage rates *κ*_*s*_ = 0 and *κ*_*s*_/*κ* = 1.0, as shown in Fig. 4. In Fig. 4, if we take the negligible side-leakage ${\kappa }_{s}({\kappa }_{s}\ll \kappa )$ and the adjusted frequencies as *ω* − *ω*_*c*_ = *κ*/2 for *g*/*κ* = 2.4, *γ*/*κ* = 0.1, and ${\omega }_{{{\rm{X}}}^{-}}={\omega }_{c}$, the reflectances, rates, and phase shifts for the reflection operator can be obtained as |*r*_h_| = |*r*_0_| ≈ 1, |*n*_h_| ≈ 0, |*n*_0_| = |*s*_h_| = |*s*_0_| = 0, and *φ*_rh_ ≈ 0, *φ*_r0_ ≈ −*π*/2, *φ*_nh_ ≈ 0, *φ*_n0_ = *φ*_sh_ = *φ*_s0_ = 0. Thus, the leaky modes ${\hat{S}}_{{\rm{i}}{\rm{n}}}$ and vacuum noise $\hat{N}$ can be ignored by choosing the parameters *g*/*κ* = 2.4 and *κ*_*s*_ = 0 with *ω* − *ω*_*c*_ = *κ*/2 and *γ*/*κ* = 0.1. After the interaction with the QD-cavity system, the reflected photon and electron spin states of the photon-electron system from Eq. 13 can be given by

14 $$|R\rangle |\uparrow \rangle \to -i|R\rangle |\uparrow \rangle ,\,|L\rangle |\uparrow \rangle \to |L\rangle |\uparrow \rangle ,\,|R\rangle |\downarrow \rangle \to |R\rangle |\downarrow \rangle ,\,|L\rangle |\downarrow \rangle \to -i|L\rangle |\downarrow \rangle .$$

In Section 3, for the reliable performance of our scheme in practice, we should calculate the fidelity F_QD_ of the interactions between a photon and QDs inside a single-sided cavity including the leaky modes ${\hat{S}}_{{\rm{i}}{\rm{n}}}$ and vacuum noise $\hat{N}$. If we consider the parameters *g*/*κ* = 2.4, *γ*/*κ* = 0.1, and *κ*_*s*_ = 0 for ${\omega }_{{{\rm{X}}}^{-}}={\omega }_{c}$ and *ω* − *ω*_*c*_ = *κ*/2, then the reflectances, rates, and phase shifts can be obtained as |*r*_h_| = |*r*_0_| ≈ 1, |*n*_h_| ≈ 0, |*n*_0_| = |*s*_h_| = |*s*_0_| = 0, and *φ*_rh_ ≈ 0, *φ*_r0_ ≈ −*π*/2, *φ*_nh_ ≈ 0, *φ*_n0_ = *φ*_sh_ = *φ*_s0_ = 0 from Eqs 1 and 12. Thus, the ideal output state $|{\psi }_{{\rm{Id}}}\rangle$ from Eq. 14 and the practical output state $|{\psi }_{{\rm{\Pr }}}\rangle$ from Eq. 13 of the photon-electron after the interaction of the QD-cavity system can be expressed as

15 $$\begin{array}{rcl}|{\psi }_{{\rm{Id}}}\rangle  & = & \frac{1}{\sqrt{2}}[\frac{-i}{\sqrt{2}}(|R\rangle |\uparrow \rangle +|L\rangle |\downarrow \rangle )+\frac{1}{\sqrt{2}}(|R\rangle |\downarrow \rangle +|L\rangle |\uparrow \rangle )],\\ |{\psi }_{{\rm{\Pr }}}\rangle  & = & \frac{1}{\sqrt{{\rm{N}}}}[\frac{({r}_{{\rm{0}}}+{s}_{{\rm{0}}})}{\sqrt{2}}(|R\rangle |\uparrow \rangle +|L\rangle |\downarrow \rangle )+\frac{({r}_{{\rm{h}}}+{n}_{{\rm{h}}}+{s}_{{\rm{h}}})}{\sqrt{2}}(|R\rangle |\downarrow \rangle +|L\rangle |\uparrow \rangle )],\end{array}$$

where the input state is $(|R\rangle +|L\rangle )/\sqrt{2}\otimes (|\downarrow \rangle +|\uparrow \rangle )/\sqrt{2}$, and ${\rm{N}}=|{r}_{{\rm{0}}}+{s}_{{\rm{0}}}{|}^{2}+|{r}_{{\rm{h}}}+{n}_{{\rm{h}}}+{s}_{{\rm{h}}}{|}^{2}$.

Subsequently, the fidelity F_QD_ between $|{\psi }_{{\rm{Id}}}\rangle$ and $|{\psi }_{{\rm{\Pr }}}\rangle$ in the QD-cavity system can be calculated as

16 $${{\rm{F}}}_{{\rm{QD}}}\equiv |\sqrt{\langle {\psi }_{{\rm{\Pr }}}|{\psi }_{{\rm{Id}}}\rangle \langle {\psi }_{{\rm{Id}}}|{\psi }_{{\rm{\Pr }}}\rangle }|=\frac{1}{\sqrt{2}}|\sqrt{\frac{{|i({r}_{{\rm{0}}}+{s}_{{\rm{0}}})+({r}_{{\rm{h}}}+{n}_{{\rm{h}}}+{s}_{{\rm{h}}})|}^{2}}{{|{r}_{{\rm{0}}}+{s}_{{\rm{0}}}|}^{2}+{|{r}_{{\rm{h}}}+{n}_{{\rm{h}}}+{s}_{{\rm{h}}}|}^{2}}}|.$$

**Figure 5:**
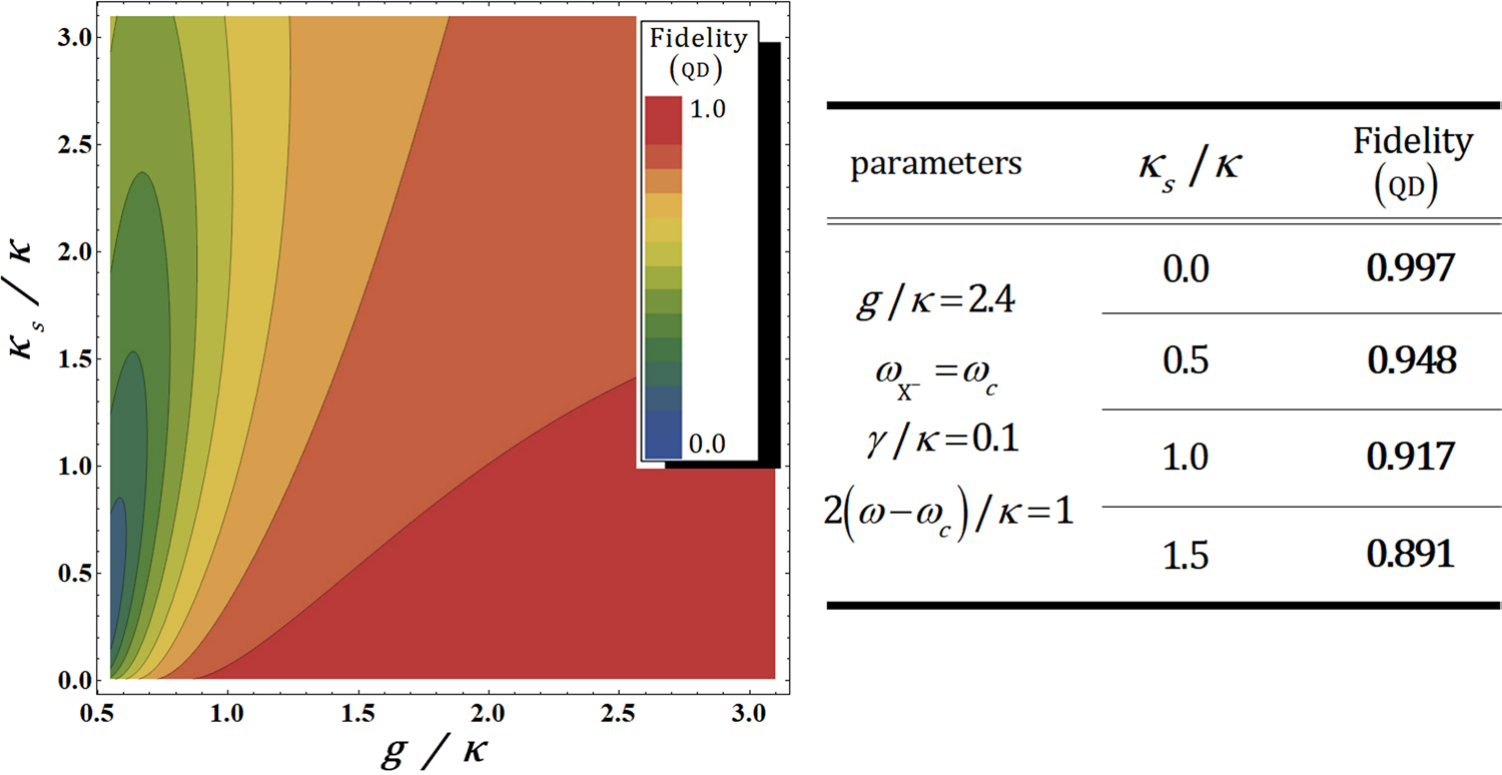
The left plot represents the fidelities F_QD_ (QD inside a single-sided cavity) of the output state with respect to the differences in side leakage rate *κ*_*s*_/*κ* and the coupling strength *g*/*κ* between the QD and the cavity with fixed *γ*/*κ* = 0.1 and ${\omega }_{{{\rm{X}}}^{-}}={\omega }_{c}$. The right table is a list of the values of F_QD_ for *κ*_*s*_/*κ* = 0.1, 0.5, 1.0, and 1.5 with fixed parameters *g*/*κ* = 2.4 and 2(*ω* − *ω*_*c*_)/*κ* = 1.

As described in Fig. 5, when the coupling strength, *g*/*κ*, is strong $(g\gg (\kappa ,\gamma ))$, and *κ*_*s*_/*κ* is the small side leakage rate $(\kappa \gg {\kappa }_{s})$ with *ω* − *ω*_*c*_ = *κ*/2 (single-sided), we can conclude that the fidelity F_QD_ of the output state approaches ‘1’, and the effect of the leaky modes ${\hat{S}}_{{\rm{i}}{\rm{n}}}$ and vacuum noise $\hat{N}$ can be omitted. Consequently, by our analysis of the QD-cavity system, we can acquire the reliable performance of the interaction between a photon and an electron in QD for the generation of hyperentanglement in our scheme.

### The polarization entangler (parity gate)

In the parity gate using XKNLs, the decoherence effect gives rise to photon loss of quantum bus beams and dephasing of coherent parameters of photon-probe systems in optical fibers^69–74,85,86^. Thus, the probability of success (P_suc_ = 1 − P_err_) and the fidelity F_XKNL_ of the output state between the ideal case and the practical case will decrease due to photon loss and dephasing (evolving quantum state to mixed state) in practice. For analysis of the influence of the decoherence effect, we introduce a master equation^87^ to describe the parity gate.

17 $$\frac{\partial \rho }{\partial t}=\hat{J}\rho +\hat{L}\rho ,\quad \hat{J}\rho =\lambda {a}\rho {{a}}^{+},\quad \hat{L}\rho =-\frac{\lambda }{2}({{a}}^{+}{a}\rho +\rho {{a}}^{+}{a}),$$

where *λ* and *t*(= *θ*/*χ*) are the energy decay rate and the interaction time in the solution $\rho (t)=\exp [(\hat{J}+\hat{L})t]\rho (0)$. Due to this solution, we can calculate the interaction of XKNL ${\tilde{X}}_{t}$ with the decoherence effect (photon loss and dephasing), ${\tilde{D}}_{t}$, by the process of ${\tilde{D}}_{t}{\tilde{X}}_{t}$ for *t*(interaction time), as follows^70,71,74^:

18 $$\begin{array}{rcl}{({\tilde{D}}_{{\rm{\Delta }}t}{\tilde{X}}_{{\rm{\Delta }}t})}^{N}|H\rangle \langle V|\otimes |\alpha \rangle \langle \alpha | & = & \exp [-{\alpha }^{2}(1-{e}^{-\lambda {\rm{\Delta }}t})\,\sum _{n=1}^{N}{e}^{-\lambda {\rm{\Delta }}t(n-1)}(1-{e}^{in{\rm{\Delta }}\theta })]\\  &  & \times |H\rangle \langle V|\otimes |{{\rm{\Lambda }}}_{t}\alpha {e}^{i\theta }\rangle \langle {{\rm{\Lambda }}}_{t}\alpha |,\end{array}$$

where we consider the divided interaction time Δ*t*(=*t*/*N*) with *N* = 10^3^ and *θ* = *χt* = *χN*Δ*t* = *N*Δ*θ* for a good approximation of our analysis [${\tilde{D}}_{t}{\tilde{X}}_{t}={({\tilde{D}}_{{\rm{\Delta }}t}{\tilde{X}}_{{\rm{\Delta }}t})}^{N}$ for *t*(=*N*Δ*t*)]. Moreover, Λ_*t*_ = *e*^−*λt*/2^ is the photon decay rate (photon loss) after the probe beam emerges from the Kerr medium. When the parity gate is implemented in practice for generating the controlled phase shift (XKNL) *θ* = *π*, the requirement for the length of the optical fiber is about 3000 km, according to *χ*/*λ* = 0.0125 (0.364 dB/km) the signal loss of commercial fibers^85,86^, and *χ*/*λ* = 0.0303 (0.15 dB/km) of pure silica core fibers^86^. Therefore, we should consider the decoherence effect (photon loss and dephasing) in the experimentally realized parity gate via our analysis. By the modeling in Eq. 18 (from the master equation) and the practical optical fiber, the output state $|{\phi }_{RR}\rangle$ in Eq. 4 of the parity gate, as described in Section 2 will evolve to a mixed state *ρ*_*RR*_, as follows:

19 $${\rho }_{{RR}}=\frac{1}{4}(\begin{array}{cccc}1 & {|{\rm{K}}|}^{2}{|{\rm{C}}|}^{2} & {|{\rm{L}}|}^{2} & {|{\rm{O}}|}^{2}{|{\rm{C}}|}^{2}\\ {|{\rm{K}}|}^{2}{|{\rm{C}}|}^{2} & 1 & {|{\rm{O}}|}^{2}{|{\rm{C}}|}^{2} & {|{\rm{L}}|}^{2}\\ {|{\rm{L}}|}^{2} & {|{\rm{O}}|}^{2}{|{\rm{C}}|}^{2} & 1 & {|{\rm{M}}|}^{2}{|{\rm{C}}|}^{2}\\ {|{\rm{O}}|}^{2}{|{\rm{C}}|}^{2} & {|{\rm{L}}|}^{2} & {|{\rm{M}}|}^{2}{|{\rm{C}}|}^{2} & 1\end{array}),$$

where the row and column of *ρ*_*RR*_ are ${|{\rm{\Lambda }}}_{t}\alpha {\rangle }^{{\rm{a}}}|HV{\rangle }_{{\rm{AB}}}|0{\rangle }^{{\rm{b}}}$, $|{{\rm{\Lambda }}}_{t}\alpha {\rangle }^{{\rm{a}}}|VH{\rangle }_{{\rm{AB}}}|0{\rangle }^{{\rm{b}}}$, $|{{\rm{\Lambda }}}_{t}\alpha \,\cos \,\theta {\rangle }^{{\rm{a}}}|VV{\rangle }_{{\rm{AB}}}|i{{\rm{\Lambda }}}_{t}\alpha \,\sin \,\theta {\rangle }^{{\rm{b}}}$, and $|{{\rm{\Lambda }}}_{t}\alpha \,\cos \,\theta {\rangle }^{{\rm{a}}}|HH{\rangle }_{{\rm{AB}}}|-i{{\rm{\Lambda }}}_{t}\alpha \,\sin \,\theta {\rangle }^{{\rm{b}}}$. The coherent parameters (C, M, L, O, and K) of the off-diagonal terms from Eq. 18 are given by^74^

20 $$\begin{array}{rcl}{\rm{C}} & = & \exp [-({\alpha }^{2}/2)\,(1-{e}^{-\lambda {\rm{\Delta }}t})\,\sum _{n=1}^{N}{e}^{-\lambda {\rm{\Delta }}t(n-1)}(1-{e}^{in{\rm{\Delta }}\theta })],\\ {\rm{M}} & = & \exp [-({\alpha }^{2}/2){e}^{-\lambda t}(1-{e}^{-\lambda {\rm{\Delta }}t})\sum _{n=1}^{N}{e}^{-\lambda {\rm{\Delta }}t(n-1)}(1-{e}^{i(n{\rm{\Delta }}\theta +\theta )})],\\ {\rm{L}} & = & \exp [-({\alpha }^{2}/2){e}^{-\lambda t}(1-{e}^{-\lambda {\rm{\Delta }}t})\sum _{n=1}^{N}{e}^{-\lambda {\rm{\Delta }}t(n-1)}(1-{e}^{in{\rm{\Delta }}\theta })],\\ {\rm{O}} & = & \exp [-({\alpha }^{2}/2){e}^{-\lambda t}(1-{e}^{-\lambda {\rm{\Delta }}t})\,(1-{e}^{i\theta })\sum _{n=1}^{N}{e}^{-\lambda {\rm{\Delta }}t(n-1)}],\\ {\rm{K}} & = & \exp [-({\alpha }^{2}/2){e}^{-\lambda t}(1-{e}^{-\lambda {\rm{\Delta }}t})\sum _{n=1}^{N}{e}^{-\lambda {\rm{\Delta }}t(n-1)}(1-{e}^{-i(n{\rm{\Delta }}\theta -\theta )})].\end{array}$$

**Figure 6:**
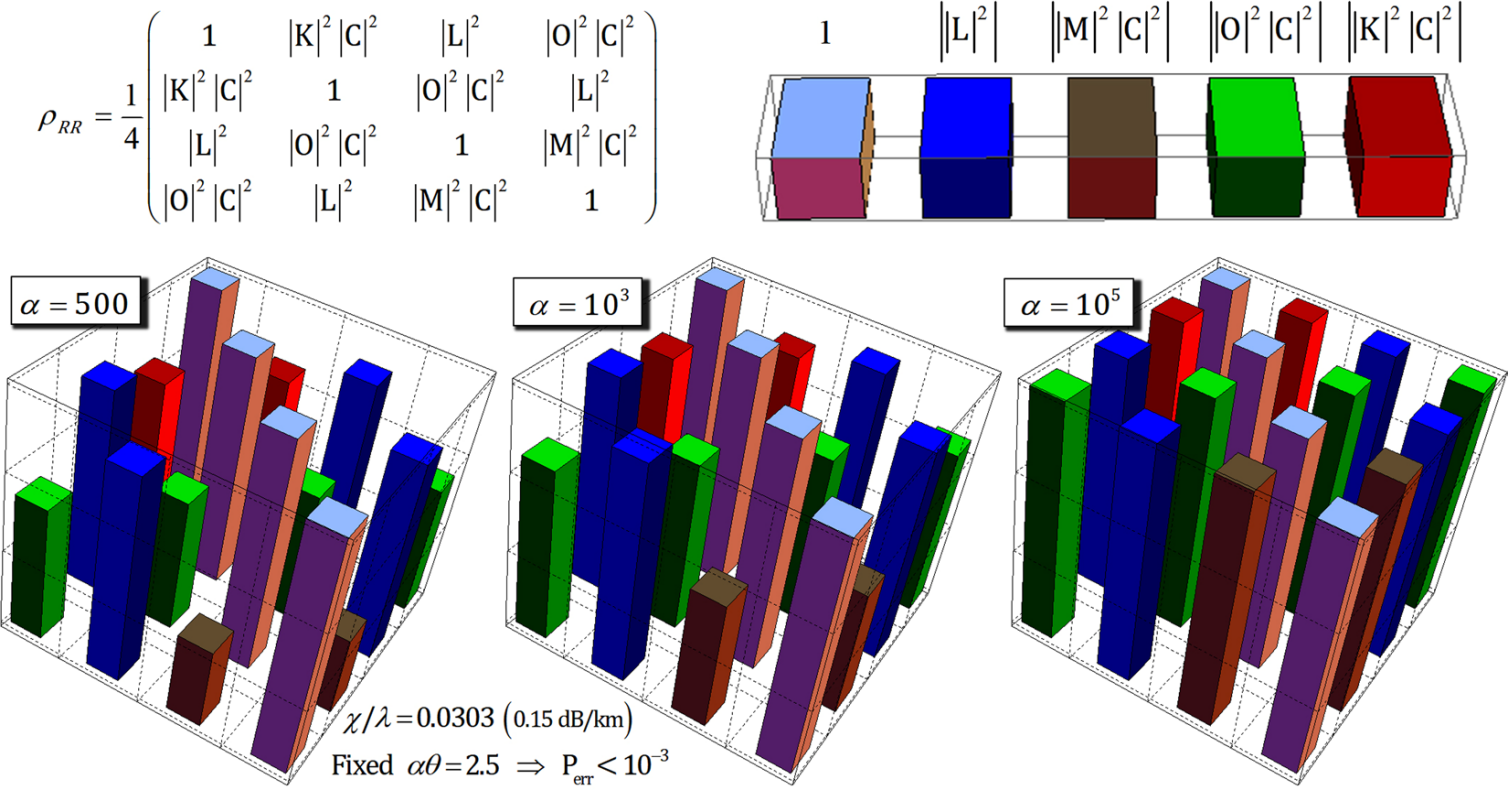
When the fixed parameter *αθ* = *αχt* = 2.5 for P_err_ < 10^−3^ and *N* = 10^3^ in the optical fiber, *χ*/*λ* = 0.0303 (0.15 dB/km), the absolute values of the coherent parameters of *ρ*_*RR*_ are plotted in diagrams depending on *α* = 500, 10^3^, and 10^5^.

Figure 6 represents the absolute values of coherent parameters (the off-diagonal terms in *ρ*_*RR*_, Eq. 19) with regard to the difference in the amplitudes of the probe beam, *α* with the fixed *αθ* = *αχt* = 2.5 for P_err_ < 10^−3^ in the optical fiber having a signal loss *χ*/*λ* = 0.0303 (0.15 dB/km)^70,71,74,86^.

In Fig. 6, we can conclude that the amplitude of the coherent state (probe beam *α*) should be increased to constrain the output state *ρ*_*RR*_ to the pure state (coherent parameters approaching ‘1’) for experimentally reliable performance of the parity gate. Thus, the reliable performance of the parity gate can be acquired by using a strong coherent state (probe beam) when the fixed *αθ* = 2.5 for P_err_ < 10^−3^ in the optical fiber, *χ*/*λ* = 0.0303 (0.15 dB/km), according to our analysis via the master equation (Eq. 17).

Due to the above result, we calculate the fidelity F_XKNL_ between the output state $|{\phi }_{RR}\rangle$, Eq. 4, in the ideal case and the output state *ρ*_*RR*_, Eq. 19, in the practical case. When we take the parameters *N* = 10^3^, *αθ* = *αχt* ≈ 2.5 for P_err_ < 10^−3^ in the optical fiber, *χ*/*λ* = 0.0303 (0.15 dB/km); the fidelity F_XKNL_ is given by

21 $${{\rm{F}}}_{{\rm{XKNL}}}\equiv |\sqrt{\langle {\phi }_{RR}|{\rho }_{RR}|{\phi }_{RR}\rangle }|=\frac{1}{2}|\sqrt{1+{|{\rm{L}}|}^{2}+{|{\rm{O}}|}^{2}{|{\rm{C}}|}^{2}+({|{\rm{K}}|}^{2}{|{\rm{C}}|}^{2}+{|{\rm{M}}|}^{2}{|{\rm{C}}|}^{2})/2}|,$$

where C, M, L, O, and K are coherent parameters (off-diagonal terms in *ρ*_*RR*_, Eq. 19) in Eq. 20.

**Figure 7:**
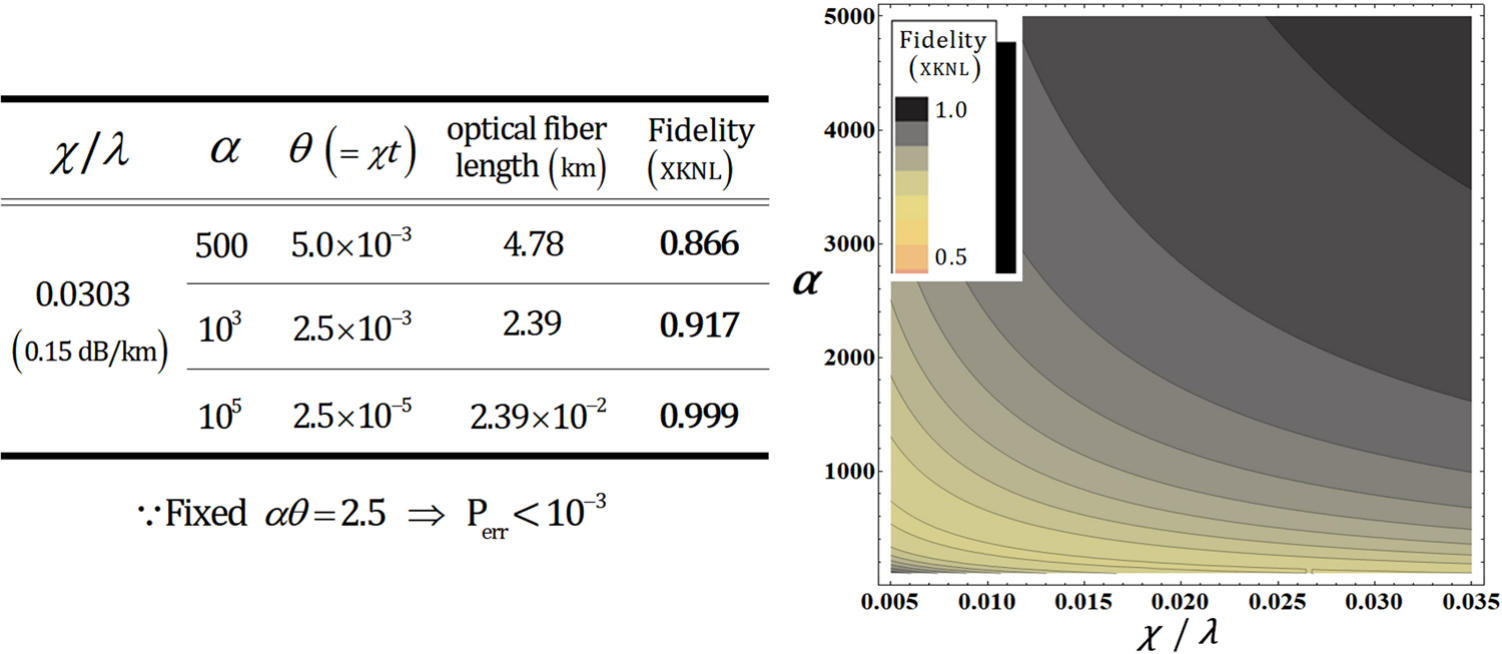
The left table is a list of the values of F_XKNL_ for *α* = 500, 10^3^, and 10^5^ in the optical fiber having *χ*/*λ* = 0.0303 (0.15 dB/km). The right plot represents F_XKNL_ of the output state with respect to differences in the amplitude of the coherent state (*α*) and the signal loss *χ*/*λ* in the optical fiber. Here we take the parameter *αθ* = 2.5 for the error probability P_err_ < 10^−3^.

Figure 7 shows that we can obtain high fidelity for the output state (F_XKNL_ → 1) by using a strong coherent state (probe beam) in the optical fiber, *χ*/*λ* = 0.0303 (0.15 dB/km)^86^ when the fixed *αθ* = 2.5 for P_err_ < 10^−3^. Furthermore, if the strong coherent state is utilized for efficient and reliable performance (high fidelity and the robustness from photon loss and dephasing induced by the decoherence effect) of the parity gate, this should decrease the magnitude of the conditional phase-shift by XKNL, as described in Fig. 7 (the left table). Therefore, we can improve the experimental feasibility of implementation of the parity gate because the natural XKNLs are extremely weak^81^. Consequently, this gate can be operated with reliable performance and the immunity from the decoherence effect for the generation of hyperentanglement in our scheme because this analysis of the parity gate using XKNLs, quantum bus beams, and the photon-number-resolving measurement.

## Conclusions

We herein propose an optical scheme to generate hyperentanglement having its own correlations for two DOFs (polarization and time-bin) on two photons using a QD-cavity system, a parity gate (XKNLs), and linear optical apparatuses (including time-bin encoders). For the reliable performance of this scheme, the most important components are two nonlinear optical devices, such as the QD-cavity system (QD in a single-sided cavity) and the polarization entangler gate (parity gate via XKNLs).

From the results of our analysis (the QD-cavity system) in Sec. 4, when the coupling strength, *g*/*κ*, is strong $(g\gg (\kappa ,\gamma ))$, and *κ*_*s*_/*κ* is the small side leakage rate $(\kappa \gg {\kappa }_{s})$ with *ω* − *ω*_*c*_ = *κ*/2 (single-sided), we can acquire high fidelity F_QD_ of the output state (photon-electron) with a negligible amount of leaky modes ${\hat{S}}_{{\rm{i}}{\rm{n}}}$ and vacuum noise $\hat{N}$. For this result, many researches have been studied, as follows: For the experimental requirements (strong coupling strength and small side leakage) in practice, Reithmaier *et al*.^33^ obtained the coupling strength $g/(\kappa +{\kappa }_{s})\approx 0.5$ in a micropillar cavity at d = 1.5 μm for the quality factor Q = 8800. When Q = 40000, increasing the coupling strength as *g*/(*κ* + *κ*_*s*_) ≈ 2.4 could be experimentally obtained as in^88^. For strong coupling, Bayer *et al*.^89^ demonstrated that micropillars with d = 1.5 μm and *γ*/*κ* ≈ 1 μeV (the decay rate of X^−^) could be acquired from In_0.6_Ga_0.4_As/GaAs (QDs) with the temperature T ≈ 2 K^89^. The side leakage rate *κ*_*s*_ can be reduced by optimizing the etching process (or improving the sample growth) with *g*/(*κ* + *κ*_*s*_) ≈ 2.4, when *g* ≈ 80 μeV and Q = 40000 (including the side leakage rate *κ*_*s*_) have been realized with In_0.6_Ga_0.4_As^79^. Moreover, the small side leakage rate can be obtained by improving the quality factor to Q = 215000 (*κ* ≈ 6.2 μeV)^90^.

In the case of the parity gate using XKNLs, for immunity (F_XKNL_ → 1) against the decoherence effect, we utilize quantum bus beams and photon-number-resolving measurement with the strong coherent state according to our analysis in Section 4. The multi-qubit gates using homodyne measurements^60,64,68^ cannot prevent evolution of the result (pure) state to a mixed state induced by the decoherence effect^69–74^, and also require a minus conditional phase-shift, −θ (which is not easy to implement due to^84^). However, our parity gate (polarization entangler) via XKNLs, quantum bus beams, and the photon-number-resolving measurement has the following advantages: **First**, the acquisition of robustness (preventing the dephasing of coherent parameters in Fig. 6) against the decoherence effect is possible to utilize the strong coherent state (probe beam) via our analysis using the master equation, as described in Section 4. **Second**, there is no requirement for the minus conditional phase-shift, −θ, which is generally known as the impossibility of changing the sign of the conditional phase-shift^84^. **Third**, the feasibility and experimental realization (the natural XKNLs are extremely weak^81^) are enhanced. This is because we can much reduce the magnitudes of the conditional phase-shifts, as listed in Fig. 7, if we employ the strong coherent state for the suppression of decoherence effect (preventing the photon loss and dephasing).

Consequently, we designed our scheme to generate hyperentanglement on two DOFs (polarization and time-bin) of two photons via the QD-cavity system, and the parity gate (polarization entangler) using XKNL, quantum bus beams, photon-number-resolving measurement, and linear optical apparatuses (time-bin encoders). Furthermore, we demonstrated by analysis the efficiency (with performance) and experimental feasibility of the nonlinear parts [QD-cavity system and parity gate (XKNLs)], which are critical components in our scheme to reliably generate hyperentanglement.
